# Motor and Predictive Processes in Auditory Beat and Rhythm Perception

**DOI:** 10.3389/fnhum.2020.578546

**Published:** 2020-09-11

**Authors:** Shannon Proksch, Daniel C. Comstock, Butovens Médé, Alexandria Pabst, Ramesh Balasubramaniam

**Affiliations:** Sensorimotor Neuroscience Laboratory, Cognitive & Information Sciences, University of California, Merced, Merced, CA, United States

**Keywords:** beat perception, motor system, motor planning, sensorimotor system, rhythm, timing

## Abstract

In this article, we review recent advances in research on rhythm and musical beat perception, focusing on the role of predictive processes in auditory motor interactions. We suggest that experimental evidence of the motor system’s role in beat perception, including in passive listening, may be explained by the generation and maintenance of internal predictive models, concordant with the Active Inference framework of sensory processing. We highlight two complementary hypotheses for the neural underpinnings of rhythm perception: The Action Simulation for Auditory Prediction hypothesis (Patel and Iversen, 2014) and the Gradual Audiomotor Evolution hypothesis (Merchant and Honing, 2014) and review recent experimental progress supporting each of these hypotheses. While initial formulations of ASAP and GAE explain different aspects of beat-based timing–the involvement of motor structures in the absence of movement, and physical entrainment to an auditory beat respectively–we suggest that work under both hypotheses provide converging evidence toward understanding the predictive role of the motor system in the perception of rhythm, and the specific neural mechanisms involved. We discuss future experimental work necessary to further evaluate the causal neural mechanisms underlying beat and rhythm perception.

## Introduction

The coupling of action and prediction in perception has been characterized by predictive models of perception (Rao and Ballard, 1999) including classical Predictive Coding (Friston, 2002, 2005) (PC), and the more recent Active Inference Framework (Active Inference – corollary to the Free Energy Principle, Friston et al., 2009; Friston, 2010; Parr and Friston, 2019). Under classical PC, the brain is thought to utilize an internal generative model and a process of probabilistic model updating to predict the causes of its sensory input. Each level of the neural hierarchy predicts the activity at the level below, with higher levels of the hierarchy providing empirical priors, or hypotheses that constrain the generation of new priors at the level below. At each level, the top-down predictive signal is compared to the bottom-up inputs from the lower level. When there is a mismatch between incoming, bottom-up sensory information and top-down predictions, a prediction error is propagated back to the level above where it is used to revise and improve the initial hypothesis. If the prediction error cannot be minimized at the level at which it is being processed, it is relayed up to the next level above. The higher in the hierarchy the prediction error is being relayed, the more substantial the revision in the hypothesis. Perceptual experience arises as prediction error is minimized and a ‘winning’ hypothesis is selected. Thus, the general idea of PC is perceptual inference.

However, this classical PC/Bayesian account of perception characterizes the brain as a passive, Helmholtzian, stimulus-response machine, responsive only to the generation of prediction errors between its top-down sensory predictions and the actual sensory input from the world (Friston and Stephan, 2007; Clark, 2013). Our brains are more aptly described as embodied and enactive, enabling us to move and interact with our environment to bring about the minimization of prediction errors through our own action (Thompson, 2007; Gallagher et al., 2013; Bruineberg et al., 2018). This is the premise of Active Inference. As in PC, the brain uses an internal generative model to predict incoming sensory data. However, rather than relying on the passive accumulation of bottom-up sensory prediction errors that are minimized to create the content of perception, Active Inference formulations incorporate active engagement with the world to make the sensory inputs more predictable. Thus, in Active Inference, the prediction error minimization process which gives rise to perceptual experience is achieved through actions which conform sensory inputs to the brain’s predictions (Friston et al., 2009; Hohwy, 2013; Parr and Friston, 2019).

Music perception and production are exemplar cognitive and behavioral phenomena to study these predictive processes and to evaluate the role of motor processing in sensory perception. Koelsch et al. (2019) expanded on the specific properties of music which make it an ideal paradigm for investigating predictive processes in the brain. Music, in any culture, is based on the generation of regularities, from the temporal regularities of rhythm to the predictable patterns and combinations of musical pitches. These regularities, or expectancies, generated by music have even been proposed as the properties which underlie emotional experience in music (Meyer, 1956; Huron, 2008; Juslin and Västfjäll, 2008). Cross-cultural perceptual priors may exist for some aspects of rhythm perception and production (Jacoby and McDermott, 2017), while other aspects are shaped by encluturation within a certain musical niche (Cameron et al., 2015; van der Weij et al., 2017; Polak et al., 2018). In particular, the experience of musical groove, that property of ‘wanting to move’ to the music, is proposed to be related to the balance between prediction and prediction errors generated by rhythmic properties of the music (Janata et al., 2012; Matthews et al., 2019, 2020). Active Inference formulations account for not only predictions related to expected stimulus input, but also predictions related to the expected accuracy–the *precision*, or uncertainty–of the original sensory prediction, in addition to counterfactual predictions related to how these prediction errors and their precision would change in response to active motor engagement with the sensory stimulus. Expected precision is modulated by sensory context and active engagement with the sensory signal. The generation of internal, predictive sensorimotor timing signals aligned to the musical beat may enhance the prediction and precision of temporal expectancies when perceiving syncopated musical rhythms, such as in musical groove (Koelsch et al., 2019). Whether or not we actually move our bodies to a musical rhythm, interactions between sensory and motor systems in our brain have been theorized to generate predictive timing signals that help us process musical rhythm (Merchant and Honing, 2014; Patel and Iversen, 2014; Vuust and Witek, 2014). These predictive timing signals are what allow for *beat induction*, or the active detection of the pulse in rhythmic time-varying stimuli such as music (Honing et al., 2014).

While predictive theories or perception are not new [indeed, they precede the age of Helmholtz, dating as far back as the 11th century works of Arab scholar al-Haytham et al. (ca. 1030; 1989)], the purpose of this review is to contextualize recent advances in the role of the motor system in rhythm and musical beat perception under more recent advances within the Active Inference framework. We then directly compare two hypotheses for the neural underpinnings of rhythm perception: The Action Simulation for Auditory Prediction (ASAP) hypothesis (Patel and Iversen, 2014) and the Gradual Audiomotor Evolution (GAE) hypothesis (Merchant and Honing, 2014). We suggest that the both hypotheses–taken together under the umbrella of Active Inference–provide converging evidence toward understanding the predictive role of the motor system within a distributed sensorimotor network underlying the perception of rhythm.

## Action and Prediction in Rhythm Perception

The role of the motor system in rhythm perception is most obviously recognized by examining how it is we engage our body with music. In addition to beat induction in passive music listening, humans – and a limited group of birds and mammals (Kotz et al., 2018; Ravignani et al., 2019) – can move in time to a musical beat. This process of rhythmic entrainment is defined as the ability to flexibly perceive and synchronize to the beat of music or other complex auditory rhythms. It is argued that rhythmic entrainment abilities are determined by the ability to perceive a beat, the underlying pulse, within rhythmic stimuli. Beat perception in humans is inherently predictive, constructive, hierarchical, and modality biased. In addition, beat perception engages the motor system, even when no movement is present (Grahn and Brett, 2007; Chen et al., 2008a,b; Gordon et al., 2018).

In humans, behavioral evidence for prediction in beat perception comes from tapping experiments that reveal negative mean asynchronies, which are not observed in other primates. Asynchronies are observed when humans tap slightly earlier or later than the beat in a rhythmic stimulus, and negative mean asynchronies are a behavioral indicator that humans actively anticipate upcoming stimuli. Mean tapping asynchronies throughout a rhythmic stimulus are usually negative in the auditory domain, but much more variable in the visual domain (Pabst and Balasubramaniam, 2018). Humans also adjust future tapping response based on temporal mismatch between their movement and the current beat (Balasubramaniam et al., 2004), and overtly tapping along to the beat aids in forming temporal predictions when compared to passively tracking a beat (Morillon and Baillet, 2017). In addition, when visual stimuli are presented in a way that indicates movement over time, e.g., apparent hand motion (Hove and Keller, 2010) or a bouncing ball (Iversen et al., 2015), predictive entrainment as demonstrated by negative mean asynchrony becomes much more successful.

According to Active Inference, the brain minimizes prediction error either by updating predictions or by taking action in the world to bring actual proprioceptive input in line with top-down predictions regarding driving sensory stimuli. In musical beat perception, this means that we either take action and *move* to the beat, or we update our predictions by suppressing actual movement and instead establishing an internal model of the beat which corresponds to the proprioceptive input we would have received had we actually been moving to the beat. The ability to flexibly adapt motor behavior in response to a mismatch between a rhythmic auditory stimulus and current motor movement (Balasubramaniam et al., 2004) can be construed as one example of this more general active inference process. Enhanced rhythmic entrainment abilities for the visual domain when visual stimuli implies movement (Hove and Keller, 2010; Iversen et al., 2015), and the improvement of temporal predictions in conjunction with overt rhythmic movement (Morillon and Baillet, 2017) can also be explained by the increase of sensory information available in order to update and modulate descending predictions about the temporal regularities of the stimulus which guide motor movements.

But this Active Inference gloss on beat perception is – by itself – vague. Plausible neural architectures have been proposed to support the classical (Helmholtzian) PC/Bayesian processing of music in general (Friston and Friston, 2013). However, an empirically detailed account of the specific neural underpinnings of *embodied Active Inference* in human musical beat perception is necessary. The motor system has been proposed to play a key role in prediction and perception of sensory information (Schubotz, 2007), and is functionally organized to enable the driving (ascending) and modulatory (descending) message passing hypothesized within the Active Inference Framework (Adams et al., 2013). This differs slightly from traditional theories of motor control, where driving signals arise from descending, top-down motor commands. Under Active Inference, top-down predictive signals from the motor system serve to modulate proprioceptive predictions regarding driving, feed-forward sensory signals (Adams et al., 2013).

Concordantly, the motor system has been found to be consistently active when listening to music, even in the absence of specific motor movement. A recent meta-analysis of fMRI studies found clusters of activations in key regions of the motor system in passive music listening, including bilateral premotor cortex and right primary motor cortex (Gordon et al., 2018). Metrical musical stimuli have also elicited activation in the basal ganglia, supplementary motor area, and cerebellum (Grahn and Rowe, 2009). Indeed, the modality bias for human beat perception and rhythmic entrainment for auditory stimuli (Pabst and Balasubramaniam, 2018), and improvements of auditory beat processing when making overt action (Morillon and Baillet, 2017) can be explained by tight connections between auditory and motor regions of the brain. But the activation of motor structures of the brain, even in the absence of overt movement, indicates that the motor system plays a more fundamental role in the formation of abstract predictive models which support sensory perception (Schubotz, 2007; Adams et al., 2013; Patel and Iversen, 2014).

Strong explanations of rhythm perception must account not only for prediction in action, but also for the role of the motor activity observed in passive music listening. Below, we provide an overview on the motor system’s role in rhythm perception, and review two complementary hypotheses which highlight the causal role of the motor system in beat-based timing perception.

## Motor System in Rhythm Perception: Views From the Action Simulation for Auditory Prediction and the Gradual Audiomotor Evolution Hypotheses

Rhythm perception involves two types of timing perception, interval-based (absolute) timing and beat-based (relative) timing (Grube et al., 2010; Ross et al., 2016a; Iversen and Balasubramaniam, 2016). Interval-based timing refers to the ability to discriminate absolute differences in interval duration, whereas beat-based timing refers to the ability to measure the duration of time intervals relative to underlying temporal regularities such as beats (Teki et al., 2011). Beat-based timing perception is thought to be uniquely human (Merchant and Honing, 2014), and is believed to rely on the formation and maintenance of internal predictive models. According to the ASAP hypothesis (Patel and Iversen, 2014), these internal predictive models consist of periodic motor planning activity communicated via the dorsal auditory stream which allow for auditory prediction in beat-based musical timing perception. ASAP highlights the dorsal auditory stream due to its structural and functional relationship between auditory and motor planning regions, facilitating temporally-precise two-way signaling between these regions. This neural pathway involved in spatial processing of sounds (Rauschecker and Tian, 2000; Patel and Iversen, 2014) is more developed in humans than non-human primates, which is consistent with differences in beat-based timing behavioral ability (Honing, 2012; Patel and Iversen, 2014). In addition, Rauschecker (2018) postulates that the dorsal auditory stream may also be forming an “internal model of the outside world…[which] conver[ts] sensorimotor sequences into a unified experience”(p264–5). In the case of musical beat-based timing perception, the dorsal stream should form an internal model of the periodic musical beat.

**FIGURE 1:**
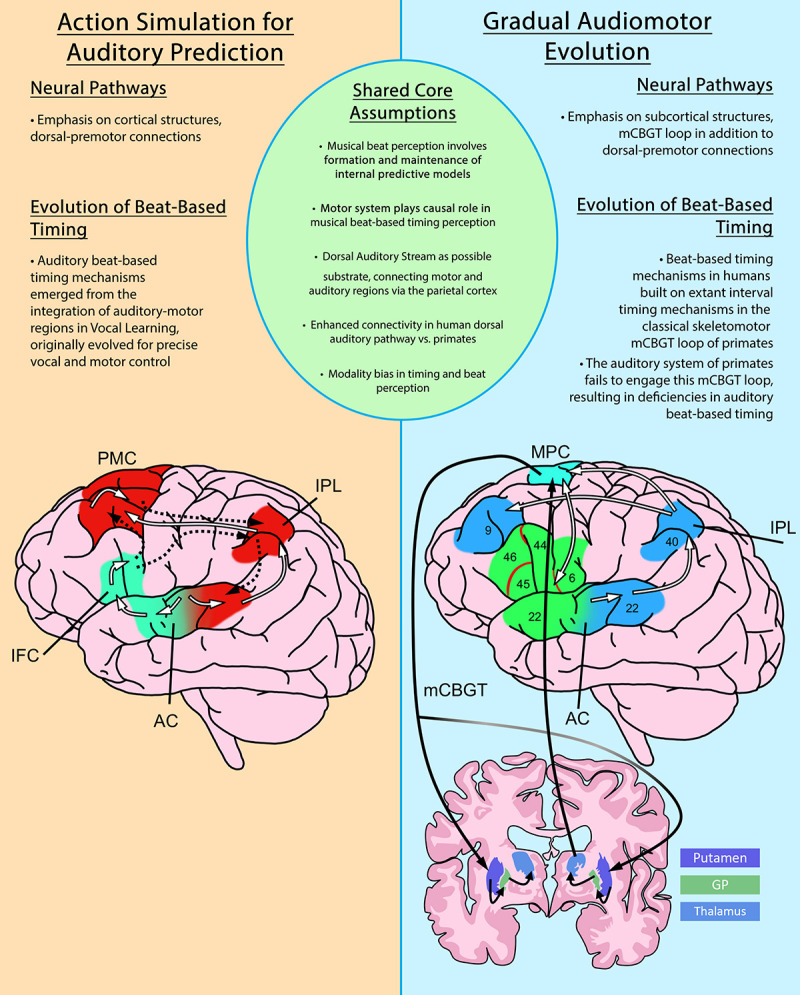
An overview comparison of the Action Simulation for Auditory Prediction Hypothesis (ASAP) and the Gradual Audiomotor Evolution Hypothesis (GAE). Shared core assumptions of both hypotheses are listed at center. Brief differing emphases on neural pathways and evolutionary commitments are listed in each panel. Diagrams depict the neural pathways proposed under each hypothesis. The ASAP diagram (left), shows ascending pathways from the auditory cortex (white lines) and descending pathways back to the auditory cortex (dashed lines) in the dorsal (red) and ventral (green) streams. The GAE diagram (right) shows the dorsal auditory pathway (white lines) and dorsal (blue) and ventral (green) streams, and the motor cortico-basal ganglia-thalamo-cortical (mCBGT) circuit (black lines). PMC, primary motor cortex; IPL, inferior parietal lobule; AC, auditory cortex; IFC, inferior frontal cortex; MPC, medial premotor cortex; GP, globus pallidus. Figures adapted from Merchant and Honing (2014) and Patel and Iversen (2014).

Complementary to the ASAP hypothesis, the GAE hypothesis has been proposed to account for differences in beat-based temporal processing between primates and humans (see Figure 1 for an overview comparison of ASAP and GAE). The GAE hypothesis (Merchant and Honing, 2014) also posits the dorsal auditory stream as a potential substrate for rhythm entrainment and perception. However, GAE claims that the evolution of rhythmic entrainment results more specifically from adaptations to the motor cortico-basal ganglia thalamo-cortical circuit (mCBGT). This specification arises from observations that the mCBGT is found to be active in sequential and temporal processing and movement in Macaques (Tanji, 2001; Merchant et al., 2013; Perez et al., 2013) and humans (Grafton et al., 1995; Harrington et al., 2010), including, for humans, the processing of musical rhythms (Grahn and Brett, 2007). Explicitly including the mCBGT loop in the evolution of rhythmic entrainment accounts for the fact that interval-timing ability appears preserved in macaques (Merchant et al., 2013) and is shared among primates, including humans. This indicates a shared neural circuitry for single interval-based timing, upon which GAE hypothesizes human beat-based timing mechanisms would have evolved to enable beat-based rhythmic entrainment. It is gradual changes to this foundational neural pathway, in addition to strengthening connections to auditory cortices via the dorsal auditory pathway, that have enabled the human mCBGT to develop beat-based timing mechanisms that can process the hierarchical properties of beat-based, rhythmic stimuli, such as music. Although focusing on slightly different neural pathways, both ASAP and GAE highlight the predictive role of the *motor system* in the perception of rhythm, and support growing consensus on the role of motor pathways in the formation of internal predictive models in perception more generally.

One important difference between the ASAP and GAE hypotheses is that ASAP purports to explain the presence of motor activity in beat perception even in the absence of overt movement, while GAE explains how evolution within motor pathways enables physical entrainment–synchronized movement–to a rhythmic stimulus. ASAP claims that beat-perception in humans arose with the emergence of *vocal learning* abilities, which strengthened tight audio-motor connections in the dorsal auditory stream underlying rhythmic entrainment along the primate lineage.

In contrast, GAE favors a gradual strengthening of these connections over evolutionary time, building on specific interval-timing mechanisms already extant in the mCGBT circuit of the primate brain. The result being the formation of an additional beat-based mechanism with enhanced connection of the mCGBT to the auditory cortex via that same dorsal auditory stream in the human brain (Merchant and Honing, 2014). Recent neurophysiological evidence highlights the interconnectedness of interval and beat-based timing mechanisms proposed by GAE, indicating that even in passive listening, monkeys are able to detect isochrony in rhythm, due in part to extant interval-based timing mechanisms of the monkey motor system, but that monkeys cannot detect the underlying beat in a rhythmic stimulus, which requires auditory-motor beat-based timing mechanisms present in humans (Honing et al., 2018).

### Evidence for Prediction and Motor Activity in GAE and ASAP

In addition to fMRI observation of motor activation in music listening and rhythm processing, the predictive and causal roles of specific motor structures highlighted by the ASAP and GAE hypotheses have been experimentally tested via electroencephalography (EEG) and transcranial magnetic stimulation (TMS). Specific Event Related Potentials (ERPs) relating to prediction errors evoked by rhythmic deviations in musical stimuli include the mismatch negativity (MMN) and P3a (Honing et al., 2018; Koelsch et al., 2019). These auditory event related components indicate violation of temporal expectations in oddball paradigms, with early responses related to bottom-up sensory processing and later responses reflecting top-down cortical processes (Garrido et al., 2007) and (perhaps conscious) attention to deviant stimuli (Sussman et al., 2003). EEG studies provide insight into the neural mechanisms of beat-perception while removing the limitations of behavioral response (Honing, 2012). The MMN and P3a components have been observed in response to rhythmic violations in adult humans, as well as infants and monkeys (Ladinig et al., 2009; Winkler et al., 2009; Honing et al., 2018). However more recent research in monkeys comparing ERPs in passive listening to jittered and isochronous stimuli with occasional deviants have demonstrated that monkeys might be able to detect isochrony in rhythm – which could rely on extant interval timing mechanisms in the primate brain; but not the beat – which relies on more evolved beat-based timing mechanisms, while humans are able to detect both isochrony and the beat (Bouwer et al., 2016; Honing et al., 2018). This collection of experiments supports the gradual evolution of beat-based timing mechanisms hypothesized by GAE.

Action Simulation for Auditory Prediction has been further supported by TMS research, demonstrating causal links between specific types of beat processing and regions of the dorsal auditory stream. A set of TMS experiments evaluated the role of the posterior parietal cortex (PPC), which is thought to serve as an interface for bidirectional communication between auditory and motor regions of the brain, and the dorsal pre-motor cortex (dPMC), which is also part of the dorsal auditory stream and is associated with movement planning and synchronization to auditory stimuli (Chen et al., 2009; Giovannelli et al., 2014). By down-regulating neural activity in left PPC according to the Huang et al. (2005) protocol, Ross et al. (2018b) showed that left PPC may be involved in one aspect of beat-based timing–phase shift detection–but not tempo detection or discrete interval discrimination. Ross et al. (2018a) down-regulated activity in left dPMC, showing that left dPMC may be involved in tempo detection, but not phase shift detection or discrete interval discrimination. Additionally, measures of Motor Evoked Potentials (MEPs) in single pulse TMS over the motor cortex have indicated that musical groove modulates cortical excitability in the motor cortex. High levels of musical groove are characterized by syncopated rhythms, enhanced energy in the bass line, and the phenomenological property of ‘wanting to move’ with the music (Janata et al., 2012; Stupacher et al., 2013; Ross et al., 2016b). High-groove music has been shown to more strongly activate the motor system (resulting in higher MEPs) when compared with low-groove music (Stupacher et al., 2013). These results indicate the bidirectionality of auditory-motor interactions, as causally down-regulating activity in the motor cortex can impair auditory perception of aspects of musical rhythm, and varying degrees of rhythmic information in auditory stimuli (i.e., syncopation and bass frequencies in musical groove) can change aspects of motor cortical function.

### Mechanisms for Timing and Rhythm Prediction

While there is growing consensus that the motor system is causally involved in timing and rhythm perception, and that the neural substrate includes cortical structures of the dorsal auditory stream and subcortical structures within the motor-cortico basal ganglia thalamo-cortical loop, the specific neural mechanisms which enable timing and rhythm perception within these substrates remains an open question. For some years, cognitive scientists have been looking for how internal timing can be instantiated by patterns of temporal stimuli via, e.g., clock-based or oscillatory mechanisms (Povel and Essens, 1985; Large and Jones, 1999). Given the amount of neuroscientific evidence pointing to a distributed timing network in the brain (Buonomano, 2014), mechanisms of entrainment to patterns of temporal stimuli have received significant attention. The striatal beat frequency model was suggested to support a clock-based mechanism based on banks of oscillators (Matell and Meck, 2000, 2004). In contrast, Large et al. (2015) describe an oscillatory model of pulse perception called Neural Resonance Theory (NRT), which provides a plausible mechanism of adaptive entrainment and beat-based timing without requiring an internal clock mechanism. According to NRT, rhythmic stimuli are encoded in sensory networks which interact with motor networks thus entraining them to the pulse frequency. Neural entrainment is induced to the pulse, even when the rhythmic stimulus itself lacks physical information at the location of the pulse–such as silences found ‘on the beat’ within syncopated rhythms–demonstrating the influence of top-down effects on pulse perception (Large et al., 2015; Tal et al., 2017). The cerebellum has also been shown to play a prominent role in absolute timing (Nozaradan et al., 2017)–but not beat-based timing–with proposed mechanisms including an oscillatory pacemaker based on regular oscillations found within the inferior olive (Ashe and Bushara, 2014), and a state-spaced based mechanism, in which the timing of a stimulus can be inferred from the state of a relevant cortical network over time (Buonomano, 2014). In various cortical areas, ramping activity of neural firing rates has been proposed as a mechanism for interval-based timing–where interval duration is encoded in the modulation of neural spiking thresholds or by varying the slope of ramping activity preceding threshold (Durstewitz, 2003). However, in the Macaque brain, ramping activity has also been implicated for relative timing in coordination with multidimensional state space models as part of a multilayer timing system involving two neural populations (Merchant et al., 2014). These two neural populations are differentially associated with absolute and relative timing, and are observed in the medial motor cortex, consistent with the proposed role of the motor system under the GAE hypothesis (Crowe et al., 2014; Merchant et al., 2014).

Continuous state-space models have also been proposed in Active Inference accounts for the generation of predictive models in action and sensory processing more generally, neurally mediated by the balance of pre- and post-synaptic activity (Friston et al., 2017) and neuronal firing rates in, e.g., medial or lateral intraparietal areas (de Lafuente et al., 2015). Striatal dopamine in particular has been proposed to code for both prediction error and certainty in response to sensory stimuli (Sarno et al., 2017) across a variety of timescales (Schultz, 2007). Dopaminergic activity also plays a role in rhythmic motor control (Koshimori and Thaut, 2018) and is responsive to rhythmic auditory stimulation (Koshimori et al., 2019), positioning dopamine as a crucial facilitator of the motor system’s role in auditory-motor interactions underlying beat-based timing perception. The motor system’s predictive role in music and rhythm perception is only one component of larger networks of sensorimotor processing, namely the dorsal auditory pathway and the mCBGT. Further experimental and computational work is necessary to determine whether and how the specific neural mechanisms of the human motor cortex processes timing information within the cortical and subcortical networks proposed by ASAP and GAE. To facilitate the generation of experimental and computational hypotheses, we have compiled an overview of recent experimental and theoretical research on the motor and distributed brain areas and mechanisms within the dorsal auditory pathway and the mCBGT–including the dopaminergic system–which are involved in the predictive processing of auditory-motor beat and rhythm perception in Table 1.

**TABLE 1:** The motor system’s predictive role in music and rhythm perception is only one component of larger networks of sensorimotor processing, namely the dorsal auditory pathway and the motor cortico-basal ganglia-thalamo-cortical circuit.

*Brain area*	Authors	Proposed role of each brain area	Experimental task and stimulus type	Type of data
*Cerebellum*	Ivry and Schlerf, 2008	Dedicated timing mechanism; coordination of movement, internal timing mechanisms involved with sub-second timing	Theoretical Paper/Review	
	Bastian, 2006	Predictive models of movement	Theoretical Paper/Review	
	Nozaradan et al., 2017	Tracking beats in rhythms with fast tempos; more prominent role in absolute timing vs. relative timing	**Passive Listening.** Auditory rhythms designed to induce a beat – syncopated and unsyncopated.	EEG
	Gordon et al., 2018	Meta-analysis of fMRI studies of recruitment of motor system during music listening	**Meta-analysis.** Various listening tasks – auditory rhythms or music.	fMRI
*Basal Ganglia*	Merchant et al., 2013	Interacts with the cortico-thalamic-striatal circuit in a context dependent manner	Theoretical Paper/Review	
	Coull and Nobre, 2008	Perceptual temporal expectation; explicit timing	Theoretical Paper/Review	
	Nozaradan et al., 2017	Tracking beats in complex rhythm sequences	**Passive Listening** Auditory rhythms designed to induce a beat – syncopated and unsyncopated.	EEG
	Grahn, 2009	Internal beat generation; more prominent role in relative vs. absolute timing	**Discrimination task, same or different judgment of two auditory stimuli.** Auditory rhythms – beat-based structure and non-beat-based structure; Accents- duration or volume accented (externally generated) or unaccented (internally generated) beats.	fMRI/Behavioral
	Grahn et al., 2011	Internal representation of auditory rhythms that support cross-modal interactions in beat perception and generation	**Discrimination task, rhythmic tempo change.** Auditory tone metronome and visual flashing metronome. Two groups: one with auditory first visual second, and the other vice versa.	fMRI/Behavioral
	Grahn and Rowe, 2009	Internal beat generation: part of cortico-subcortical network involved in beat perception and generation	**Indicate the strength of the perceived beat.** Auditory rhythms of varying complexity and some with volume accents.	fMRI/Behavioral
	Grahn and Rowe, 2013	Putamen activity in beat prediction, but not beat finding	**Attentive listening; occasionally indicate level of feeling of the beat.** Auditory rhythms of varying intervals and rates, beat and non-beat (jittered) rhythms.	fMRI/Behavioral
	Grahn and Brett, 2007	Higher activity for rhythms with integer ratio relationships between intervals and with regular perceptual accents	*1st experiment (behavioral)* **reproduce auditory rhythms.** *2nd experiment (fMRI)* **indicate if the rhythm played matched previous rhythms**. Metered auditory rhythms of varying integer intervals and complexity.	fMRI/Behavioral
	Teki et al., 2011	Striato-thalamo-cortical network involved in beat-based timing, while an olivocerebellar network involved in duration-based timing	**Judge duration matches in a set of tones.** Auditory tones, either isochronous or jittered, arranged in either rhythm-based or absolute duration-based sets.	fMRI/Behavioral
	Araneda et al., 2017	Hearing, feeling or seeing a beat recruits a supramodal network in the auditory dorsal stream	**Discrimination task, between beat and non-beat rhythms**. Auditory, visual, and vibrotactile rhythms.	fMRI/Behavioral
*Primary and premotor cortices*	Kilavik et al., 2014	Movement preparation, cue anticipation	Theoretical Paper/Review	
	Schubotz, 2007	Predictive processing of external events, even in the absence of proprioceptive or interoceptive information	Theoretical Paper/Review	
	Morillon and Baillet, 2017	Beta and delta oscillations directed to auditory cortex encode temporal predictions	**Passive listening (listen condition); active tapping with the beat (tracking condition).** Auditory melody – different tones either on beat, anti-phase, or quasi-phase with the beat.	MEG/Behavioral
	Gordon et al., 2018	Meta-analysis of fMRI studies of recruitment of motor system during music listening	**Meta-analysis.** Various listening tasks – Auditory rhythms or music.	fMRI
*Premotor cortex*	Grahn and Rowe, 2009	Cortico-cortical coupling with SMA and auditory cortex in duration beat perception; part of cortico-subcortical network involved in beat perception and generation	**Indicate the strength of the perceived beat.** Auditory rhythms of varying complexity and some with volume accents.	fMRI/Behavioral
	Teki et al., 2011	Striato-thalamo-cortical network involved in beat-based timing, while an olivocerebellar network involved in duration based timing	**Judge duration matches in a set of tones.** Auditory tones, either isochronous or jittered, arranged in either rhythm-based or absolute duration-based sets.	fMRI/Behavioral
	Chen et al., 2008a	Motor regions recruited while listening to music rhythms	*Experiment 1:* **Listen to rhythm passively then tap along with rhythm.** *Experiment 2:* **Listen to rhythm passively then tap along to rhythm without foreknowledge of being asked to tap with the rhythm** auditory tones in simple, complex, or ambiguous rhythms.	fMRI/Behavioral
*Supplementary motor area*	Coull et al., 2016b	Perceptual and motor timing; Comparing the duration of perceptual events, error monitoring	Theoretical Paper/Review	
	Ross et al., 2018b	Not causally implicated in perceptual auditory interval timing	**Discrimination task – same/different judgment of auditory intervals; detection task – identification of tempo or phase shifted metronome click.** Auditory intervals of pairs of tones; metronome click track over musical stimuli.	Behavioral (pre/post TMS down-regulatory stimulation)
	Grahn and Brett, 2007	Higher activity for rhythms with integer ratio relationships between intervals and with regular perceptual accents; in musicians: higher activity for all rhythms when compared to rest	*Experiment 1 (behavioral):* **reproduce auditory rhythms.** *Experiment 2 (fMRI):* **indicate if the rhythm played matched previous rhythms.** Metered auditory rhythms of varying integer intervals and complexity.	fMRI/Behavioral
	Grahn and McAuley, 2009	Stronger activity in strong beat-perceivers vs. weak beat-perceivers, no correlation with musicianship	**Discrimination task, rhythmic tempo change.** Auditory isochronous rhythms.	fMRI/Behavioral
	Grahn and Rowe, 2009	Coupling with STG in beat perception for musicians; part of cortico-subcortical network involved in beat perception and generation	**Indicate the strength of the perceived beat.** Auditory rhythms of varying complexity and some with volume accents.	fMRI/Behavioral
	Teki et al., 2011	Striato-thalamo-cortical network involved in beat-based time, while an olivocerebellar network involved in duration-based timing	**Judge duration matches in a set of tones.** Auditory tones, either isochronous or jittered, arranged in either rhythm-based or absolute duration-based sets.	fMRI/Behavioral
	Chen et al., 2008a	Motor regions recruited while listening to music rhythms	*Experiment 1:* **Listen to rhythm passively then tap along with rhythm.** *Experiment 2:* **Listen to rhythm passively then tap along to rhythm without foreknowledge of being asked to tap with the rhythm** auditory tones in simple, complex, or ambiguous rhythms.	fMRI/Behavioral
	Araneda et al., 2017	Hearing, feeling or seeing a beat recruits a supramodal network in the auditory dorsal stream	**Discrimination task, between beat and non-beat rhythms.** Auditory, visual, and vibrotactile rhythms.	fMRI/Behavioral
*Medial premotor cortex*	Merchant et al., 2014	Absolute and relative timing mechanisms within two separate neural populations	Theoretical Paper/Review	
	Crowe et al., 2014	Absolute and relative timing mechanisms within two separate neural populations	**Synchronization Continuation Task.** Isochronous visual stimuli or auditory tones.	Behavioral; Extracellular activity of single neurons (in Macaca mulatta)
	Grahn and McAuley, 2009	Stronger activity in strong beat-perceivers vs. weak beat-perceivers, no correlation with musicianship	**Discrimination task, rhythmic tempo change.** Auditory isochronous rhythms.	fMRI/Behavioral
*Parietal Cortex*	Rauschecker, 2011; Merchant and Honing, 2014; Patel and Iversen, 2014	Interface between motor and auditory cortices, sensorimotor integration	Theoretical Papers/Reviews	
	Coull and Nobre, 2008	Perceptual temporal expectation; implicit timing	Theoretical Paper/Review	
	Coull et al., 2016a	Temporal predictability via fixed or dynamic predictions	**Cued reaction time task.** Visual cue that predicted target presentation time (temporal condition), or provided no information for target presentation (neutral condition) with variable intervals between cue and target.	fMRI/Behavioral
	Ross et al., 2018b	Causally implicated in perceptual beat-based timing	**Discrimination task – same/different judgment of auditory intervals; detection task – identification of tempo or phase shifted metronome click.** Auditory intervals of pairs of tones; metronome click track over musical stimuli.	Behavioral (pre/post TMS down-regulatory stimulation)
*Auditory Cortex*	Koelsch et al., 2019	Event related potentials associated with predictive processes in music	Theoretical Paper/Review	
	Fujioka et al., 2012	Beta-band activity predicts onset of beats in music	**Passive listening, while watching silent videos**. Auditory isochronous rhythms of several tempos and one irregular rhythm.	MEG
	Fujioka et al., 2015	Beta-band activity represents timing information being translated for auditory-motor coordination	**Passive listening** to metered rhythms, followed by **attentive listening** to un-metered rhythms that the participants were asked to imagine as metered. March and Waltz metered rhythms	MEG
	Auksztulewicz et al., 2019	Temporal prediction of rhythm and beats	**Identify target chords.** Auditory rhythmic or jittered sequences of distractor chords preceding target chords.	MEG/EEG/Behavioral
	Honing et al., 2018	Event related potentials to perceptual deviants in rhythmic stimuli	**Passive listening.** Auditory oddball paradigm with isochronous or jittered rhythms.	EEG (of Macaca mulatta)
	Bouwer et al., 2016	Event related potentials to perceptual deviants in rhythmic stimuli; ERPs modulated by attention in musicians	**Passive or attentive listening.** Auditory oddball paradigm with isochronous or jittered rhythms.	EEG
*Dopaminergic System/Striatal Dopamine*	Schultz, 2007	Multiple time courses of dopamine changes mediate multiple time courses of behavioral processes	Theoretical Paper/Review	
	Friston et al., 2009	Reward learning, encoding of precision	Theoretical Paper/Review	
	Friston et al., 2012; FitzGerald et al., 2015	Reward learning, encoding of precision	Theoretical Papers/Computational Models	Simulated dopaminergic responses
	Sarno et al., 2017	Temporal expectation of perceptual cues; reward prediction error and (un)certainty	**Detect weak vibrotactile stimuli.** Variable interval durations between tactile start cue and vibrotactile stimuli.	Intracellular recording, monkey brain
	Koshimori et al., 2019	Rhythmic auditory stimulation (RAS) attenuates dopaminergic response	**Synchronization task,** RAS and no-RAS conditions; various auditory rhythms, single auditory beats or metronome clicks over instrumental music.	Behavioral/MRI/PET
	Brodal et al., 2017	Rhythmic music reduces connectivity between basal ganglia and reward system	**Passive listening.** Electronic dance music in a continuous-stimulation design.	fMRI

*This table provides an overview of the brain areas and mechanisms which make up these networks and are involved in the predictive processing of auditory beat and rhythm perception. Each brain area is introduced with one or more Theoretical or Review Papers contextualizing that brain area’s proposed role, followed by a non-exhaustive list of supporting experimental work. This table is intended to serve as a tool for new or continuing researchers engaging in work on rhythm and musical beat perception.*

## Conclusion and Future Directions

In this paper, we reviewed recent advances in research on rhythm perception, focusing on the role of predictive processes in auditory motor interactions in beat-processing. We highlighted two complementary hypotheses for the neural underpinnings of rhythm perception: The ASAP hypothesis (Patel and Iversen, 2014) and the GAE hypothesis (Merchant and Honing, 2014) and reviewed recent experimental progress supporting each of these hypotheses. While initial formulations of ASAP and GAE explain different aspects of beat-based timing–the involvement of motor structures in the absence of movement, and physical entrainment to an auditory beat respectively–both theories have moved us closer to understanding the predictive role of the motor system in the perception of rhythm and the specific neural mechanisms involved. In fact, recent computational formulations of ASAP have further incorporated the subcortical structures proposed to be involved in the evolution of beat-based timing perception by GAE. Cannon and Patel(2019, preprint), have proposed the CBGT loop as responsible for the resetting of relative timing mechanisms via a hyper direct pathway from the SMA. In addition, they hypothesize a role for striatal dopamine in the maintenance of internal rhythmic timing models by tracking confidence (uncertainty) in the beat, consistent with Predictive Coding and Active Inference accounts of rhythm perception and perception more generally.

Future work in understanding the neural, cognitive, and behavioral dynamics of musical beat perception in humans should investigate not only the sensorimotor processes responsible for the perception of rhythm, but also the specific neural mechanisms by which top-down predictions serve to modulate driving proprioceptive sensations arising from concrete actions of the body or abstract activity of the motor systems. While EEG experiments (e.g., Ladinig et al., 2009; Winkler et al., 2009; Honing et al., 2018) point to the neural mechanisms of internal predictive models in beat-based timing perception, EEG alone cannot provide causal evidence for the role of specific brain structures. Similarly, while TMS experiments (e.g., Stupacher et al., 2013; Ross et al., 2018a,b) have lended causal evidence for the role of specific structures in beat-based timing perception, the mentioned experiments do not provide direct evidence for the presence of *internal predictive models* of beat-based timing. If motor activity is causally involved in the formation of auditory predictions, then causal TMS manipulation to down-regulate activity in, e.g., parietal cortex or dPMC should result in the reduction of MMN and P3a event related responses to perceptual deviants in rhythmic stimuli, and this response might differ based on whether the stimuli contains timing deviants related to tempo or phase. Future research should include stimuli designed to elicit specific prediction errors with perceptual deviants, such as in oddball paradigms, while measuring event-related potentials associated with predictive processes in combined EEG and causal TMS experiments. Results from these experiments could extend and strengthen already emerging support for GAE and ASAP, as well as further contextualize the role of Active Inference in music and beat-based timing perception.

## Author Contributions

SP and RB conceptualized the manuscript. BM, DC, and AP contributed to the writing and the exhaustive analysis of the literature. All authors contributed to the article and approved the submitted version.

## Conflict of Interest

The authors declare that the research was conducted in the absence of any commercial or financial relationships that could be construed as a potential conflict of interest.
